# 0404 inhibits hepatocellular carcinoma through a p53/miR-34a/SIRT1 positive feedback loop

**DOI:** 10.1038/s41598-017-04487-x

**Published:** 2017-06-30

**Authors:** Caixia Xia, Liyan Shui, Guohua Lou, Bingjue Ye, Wei Zhu, Jing Wang, Shanshan Wu, Xiao Xu, Long Mao, Wanhong Xu, Zhi Chen, Yanning Liu, Min Zheng

**Affiliations:** 1 The State Key Laboratory for Diagnosis and Treatment of Infectious Diseases, The First Affiliated Hospital of School of Medicine, Zhejiang University, Collaborative Innovation Center for Diagnosis and Treatment of Infectious Diseases, Hangzhou, China; 2 Department of Infectious Diseases, Hangzhou First People’s Hospital, Nanjing Medical University, Hangzhou, China; 3 Hangzhou ACEA Pharmaceutical Research Co., Ltd., Hangzhou, China

## Abstract

DNA-damaging agents have been used in cancer chemotherapy for a long history. Unfortunately, chemotherapeutic treatment strategies against hepatocellular carcinoma (HCC) are still ineffective. We screened a novel DNA-damaging compound, designated as 0404, by using time-dependent cellular response profiling (TCRP) based on unique DNA-damage characteristics. We used human HCC cell lines and HCC xenograft mouse model to analyze the anti-cancer effects of 0404. Transcriptome and miRNA arrays were used to verify the anti-cancer mechanism of 0404. It was confirmed that p53 signaling pathway was crucial in 0404 anti-cancer activity and the expression of miR-34a, a key tumor-suppressive miRNA, was up-regulated in 0404-treated HepG2 cells. MiR-34a expression was also down-regulated in HCCs compared with corresponding non-cancerous hepatic tissues. We further identified the mechanisms of 0404 in HepG2 cells. 0404 increased miR-34a expression and acylation p53 protein levels and decreased SIRT1 protein levels in a concentration-dependent manner. The sensitivity of HepG2 cells to 0404 was significantly decreased by transfection with miR-34a inhibitors and SIRT1 protein levels were up-regulated by miR-34a inhibition. Our findings show that 0404 is probably an attractive agent for treating HCC, especially in HCC with wide type (WT) p53, through forming a p53/miR-34a/SIRT1 signal feedback loop to promote cell apoptosis.

## Introduction

Hepatocellular carcinoma, a common malignant tumor, is recognised as the third most common cause for cancer-related mortality^1, 2^ due to a lack of effective treatment options. Currently, the treatment of HCC is highly depending on the systemic therapy with classical chemotherapy as most patients presented at a stage when surgical resection was no longer possible. Nevertheless, systemic chemotherapy is reported to show poor tolerance and very low response^3^. Thus, it is urgent to develop new chemotherapeutic drugs for HCC^4^.

Cancer cells, with the capacity to sense and repair DNA damage, can escape from cell-cycle checkpoints, allowing cellular proliferation in an unlimited manner. In turn, the high proliferation rate may induce increased susceptibility of cancer cells to DNA damage as the replication of damaged DNA contributed to cell death^5^. Nowadays, numerous anti-cancer agents such as cisplatin, doxorubicin and 5-fluorouracil have been developed with DNA-damaging as a target for anti-cancer therapy. DNA-damaging compounds have a long history of use in chemotherapy of various cancers^6, 7^, including breast cancers, lung cancers, prostatic cancers and bladder cancers. However, the efficiency of these drugs in the management of HCC is still not satisfactory.

Nowadays, drug discovery has been reported to undergo a paradigm change from biochemical based technique to cell-based screening method. Up to now, the time-dependent cell response profiling approach^8, 9^ has been developed in the drug development to predict the mechanism of compound actions among drugs. The approach is time-dependent and participates a combined measuration of cell counts, adhesion and morphology. Moreover, such method addresses the limitation that previously only one time point is selected to evaluate the compounds’ effects and the relational mechanisms. As a tool allowing for an unbiased phenotypic screening, its main function includes identifying novel activities, as well as “off-target effects” for drugs and experimental compounds^9^. These lead us to screen a potential drug for the treatment of HCC using the TCRP approach based on a unique DNA-damaging signature.

In this study, an effective DNA-damaging compound, designated as 0404, was screened, which could induce apoptosis effectively in HCC cells at nM concentration. Besides, we investigated the anti-cancer effects of 0404 and the specific mechanisms.

## Result

### 0404 is screened as a DNA-damaging compound using TCRP

**Figure 1:**
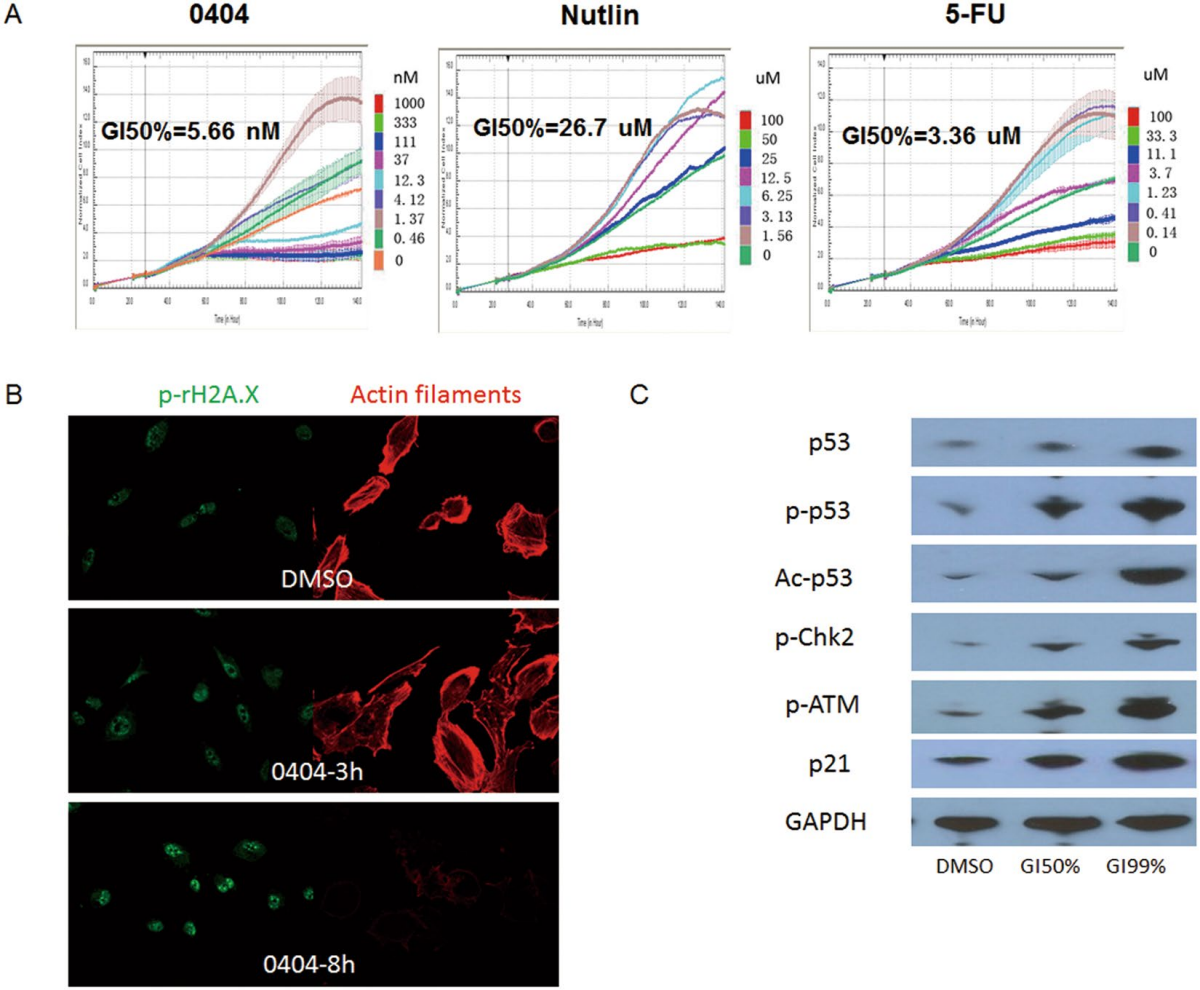
Screening of 0404 as a new DNA-damaging compound using TCRP. (**A**) 0404 was more effective than classical DNA-damaging agent as it could induce significant growth inhibition of H292 cells at nM concentration. (**B**) 0404 up-regulated p-γH2A.X level in a time-depended manner. (**C**) The expression of DNA damage-related signaling molecules was significantly up-regulated in 0404-treated A549 cells in a concentration-dependent manner.

Using the TCRP approach based on a unique DNA-damaging TCRP signature^9^, we have screened as a novel DNA-damaging compound, designated as 0404. Compared with the classical DNA-damaging agent (e.g. Nutlin-3 and 5-FU), 0404 induced significant growth inhibition in H292 cells at a nM concentration (Fig. 1A). Immunofluorescent staining showed that 0404 could up-regulate the phosphorylation of H2A.X that played a central role in sensing and repairing DNA damage in A549 cells in a time-depended manner (Fig. 1B). Moreover, Western blotting analysis showed that the expression of DNA damage-related signaling molecules was significantly up-regulated in 0404-treated A549 cells, including p53, acetylated p53 (Ac-p53), phosphorylated p53 (p-p53), phosphorylated Chk2 (p-Chk2), phosphorylated ataxia telangiectasia mutated kinase (p-ATM) and p21. Meanwhile, the up-regulation also presented in a dose-dependent manner (Fig. 1C).

### The anti-cancer effects of 0404 depend on p53 expression

**Figure 2:**
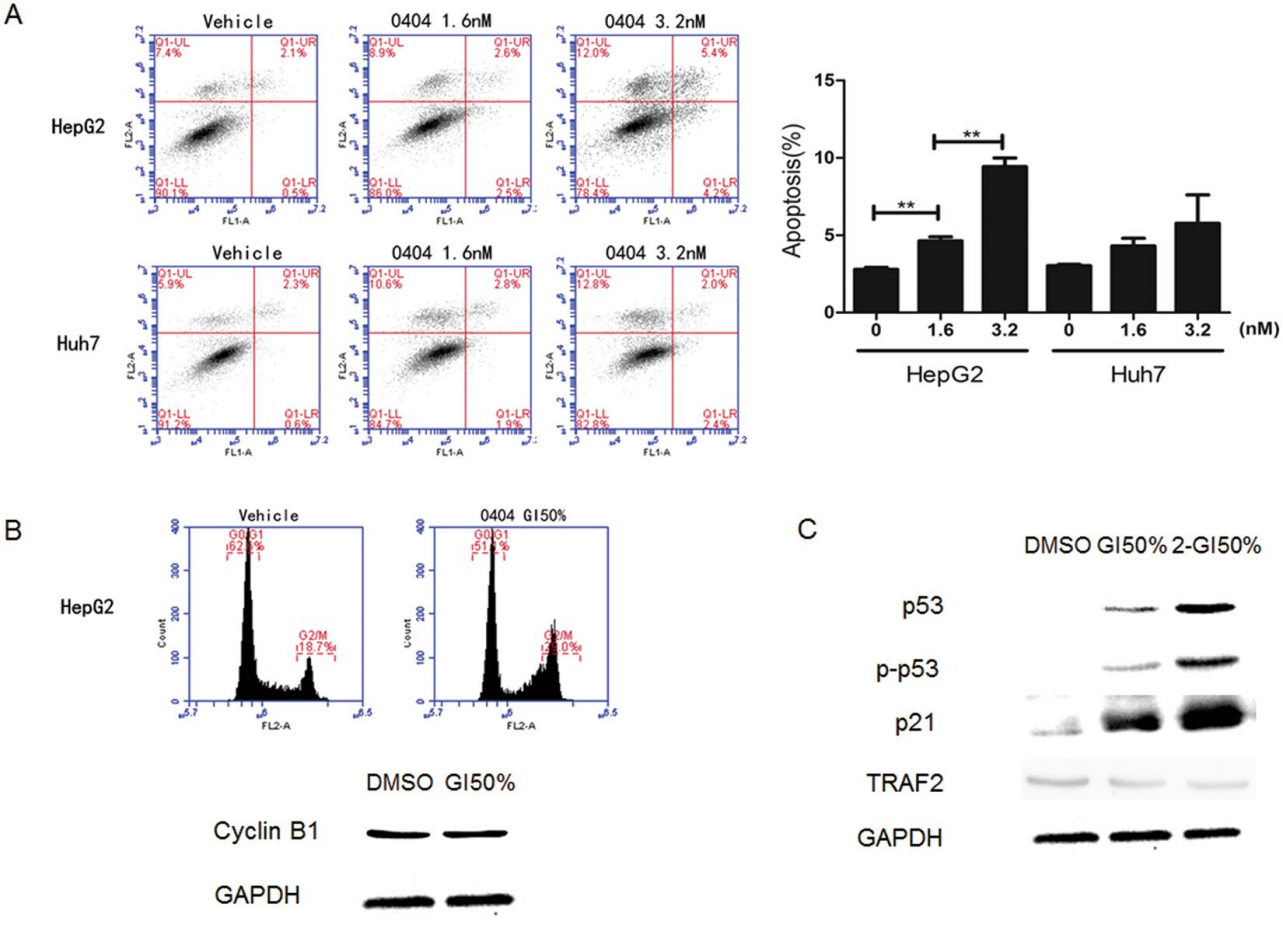
Anti-cancer effects of 0404. (**A**) 0404 inducing apoptosis of HepG2 cells was highly depending on the concentration. (**B**) The majority of HepG2 cells were arrested at the G2/M phase at GI50% concentration and cyclinB1 was decreased at the same time. (**C**) 0404 induced up-regulation of p53, the phosphorylated p53 protein, the p53 targets such as p21 and down-regulation of TRAF2 protein in HepG2 cells.

We tested the anti-cancer effects of 0404 on HepG2 (p53 WT) and Huh7 (p53 mutation (Mut)) cells. The GI50% concentration of 0404 in HepG2 cells was significantly lower than that in Huh7 cells (1.6 ± 0.1 nM v.s. 4.6 ± 0.4 nM, P < 0.05). 0404 of GI50% and 2-fold GI50% concentrations could induce obvious apoptosis of HepG2 cells in a dose-dependent manner. However, at the same concentration, no significant cell apoptosis was induced in Huh7 cells (Fig. 2A). Flow cytometry indicated that a majority of HepG2 cells were arrested at G2/M phase at a GI50% concentration, and the expression of cycle arrest related protein cyclinB1 was decreased (Fig. 2B).

We also determined the expression of p53 and the phosphorylated p53 protein in HepG2 cells, which revealed their expression was up-regulated after 0404 treatment. In addition, the expression of p53 targets (e.g. p21) was also up-regulated in a dose-dependent manner and the expression of TNF receptor-associated factor 2 (TRAF2), acting as a regulator of p53 activity and stability^10^, was down-regulated accordingly (Fig. 2C).

### The 0404 compound inhibits hepatocellular tumor growth *in vivo*

**Figure 3:**
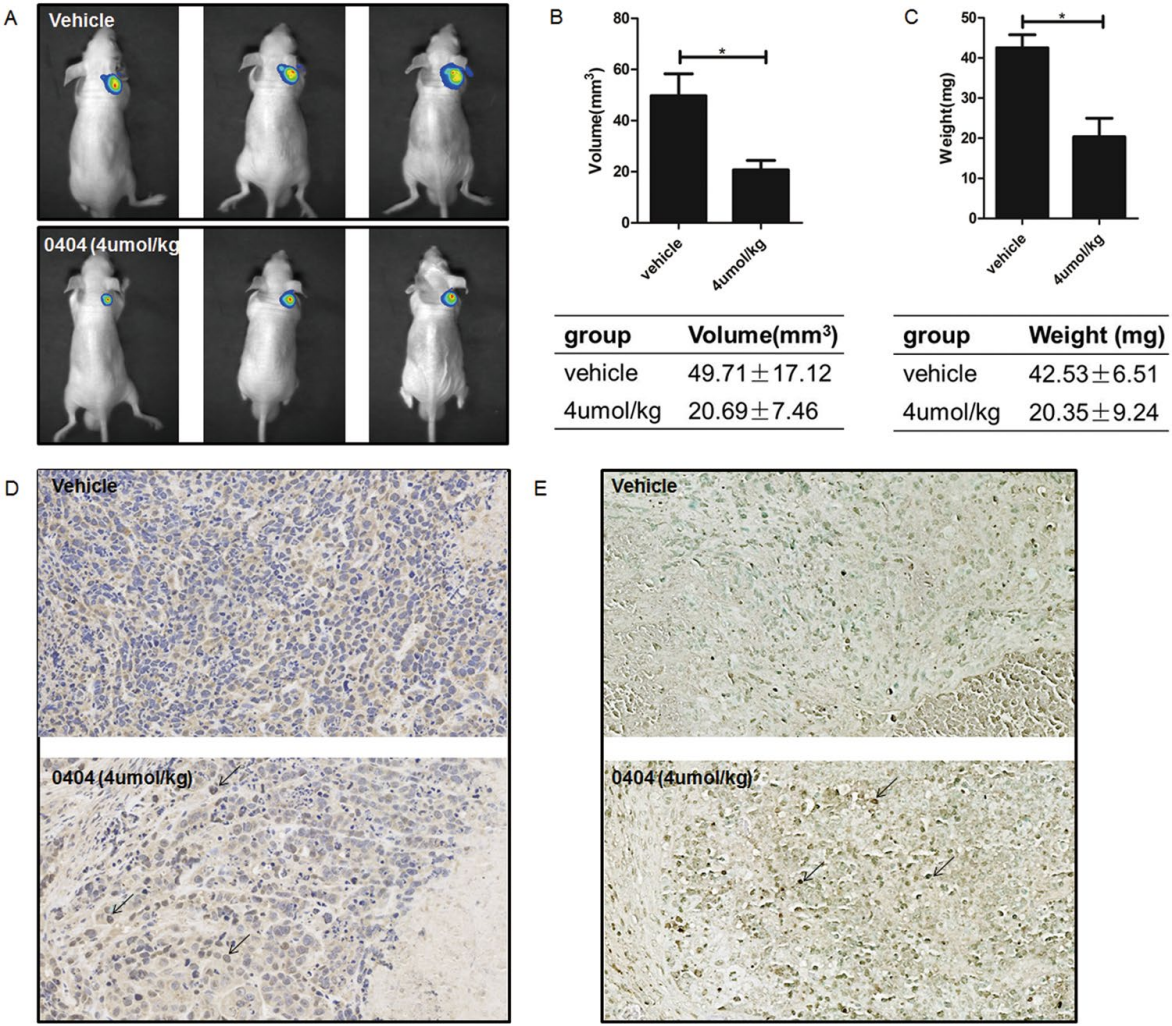
0404 inhibited hepatocellular tumor growth *in vivo*. (**A**) Luciferase emission showed that 0404 inhibited the tumor growth *in vivo*. (**B** and **C**) 0404 significantly inhibited the tumor volume and weight excised from nude mice at day 25. (**D**) p53 levels were up-regulated in the HepG2 tumors excised from nude mice after treatment with 0404. (**E**) TUNEL assay was used to detect the cell apoptosis in the HepG2 tumors excised from nude mice after treatment with 0404.

In this part, we assessed the growth of HepG2-tumor bearing nude mice mediated by 0404. Tumor growth was monitored through the measurement of luciferase emission biweekly (Fig. 3A), which revealed the tumor volume and weight were significantly inhibited at the experimental end point (Fig. 3B and C). Compared to the vehicle group, significant growth retardation was induced by 4 μmol/kg 0404 at day 25, indicating 0404 was an effective anti-HCC agent *in vivo*. What’s more, p53 level was up-regulated in the HepG2 tumors excised from nude mice after treatment with 0404 (Fig. 3D). This validated that p53 played a crucial part in the anti-cancer effects of 0404 *in vivo*. Transferase-mediated deoxyuridine triphosphate-biotin nick end labeling (TUNEL) staining was used to determine the apoptosis of HepG2 tumors induced by 0404. The results indicated the compound induced remarkable apoptosis compared to the control group (148/area vs. 19/area, Fig. 3E).

### Transcriptome and miRNA arrays verify the anti-cancer mechanism of 0404

**Figure 4:**
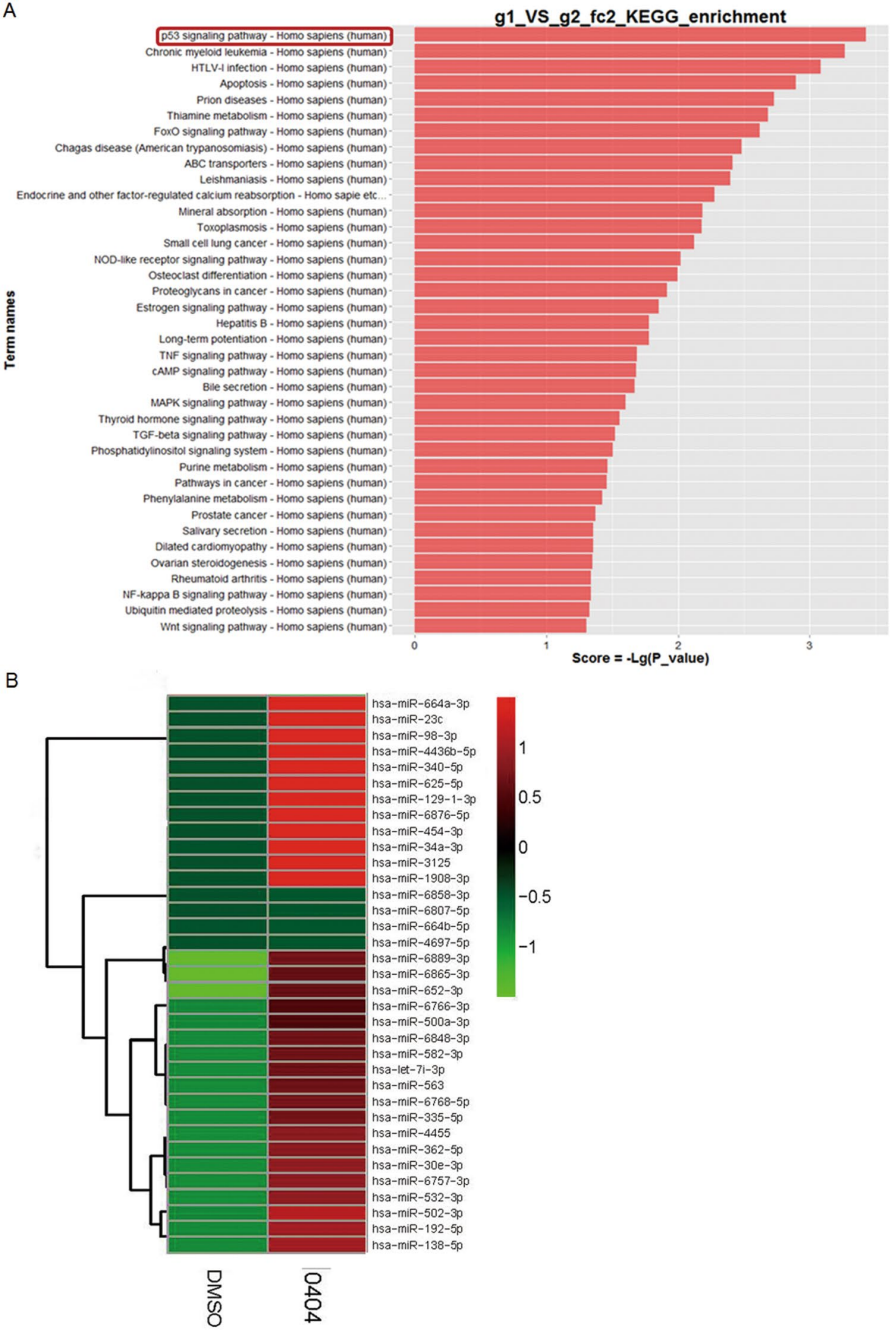
The results of 0404 in transcriptome and miRNA arrays. (**A**) The p53 signaling pathway played the key role in 0404 anti-cancer activity. (**B**) MiR-34a level showed an up-regulation in 0404-treated HepG2 cells.

To further analyze the inhibitive roles of 0404 in HCC, transcriptome and miRNA array analyses were performed in HepG2 cells. Ingenuity pathway analysis based on human transcriptome array data confirmed that the p53 signaling pathway was closely involved in 0404 anti-cancer activity (Fig. 4A). Moreover, miRNA microarray analysis showed that the expression of several tumor-suppressive miRNAs, such as miR-34a, miR- 664a and miR-340, was up-regulated in 0404-treated HepG2 cells (Fig. 4B). Among them, miR-34a was reported to be related with p53 activation^11, 12^. These data suggest 0404 may inhibit HCC growth by p53/miR-34a signaling pathway.

### Down-regulation levels of miR-34a in HCC patients

**Figure 5:**
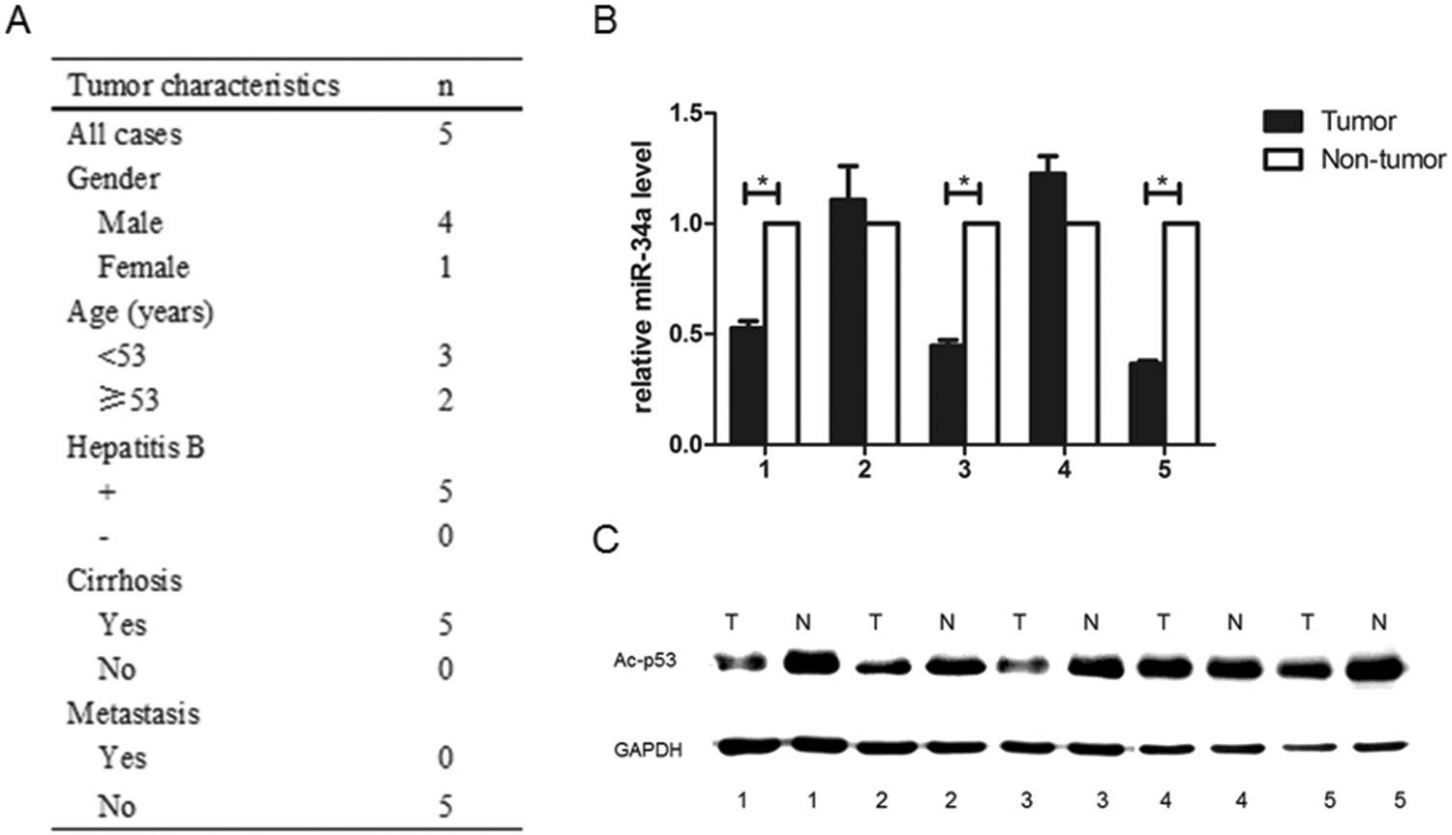
The levels of miR-34a in HCC patients were down-regulated. (**A**) The basic characteristics about the 5 HCC tissues. (**B**) Three out of five HCCs (60%) had decreased miR-34a levels as compared with corresponding non-cancerous hepatic tissues. (**C**) Ac-p53 expression was down-regulated in HCC tissues.

As previously described, miR-34a could increase p53 acetylation and transcription, which finally resulted in apoptosis^13^. We then studied the expression of miR-34a and Ac-p53 in five HCC tissues and the adjacent non-cancerous liver tissues (Fig. 5A). As shown in Fig. 5B, the expression of miR-34a was down-regulated in 3 out of 5 HCCs (60%) compared with corresponding non-cancerous hepatic tissues. Besides, Ac-p53 expression was down-regulated in HCC tissues (Fig. 5C).

### MiR-34a involves in the anti-cancer effects of 0404

**Figure 6:**
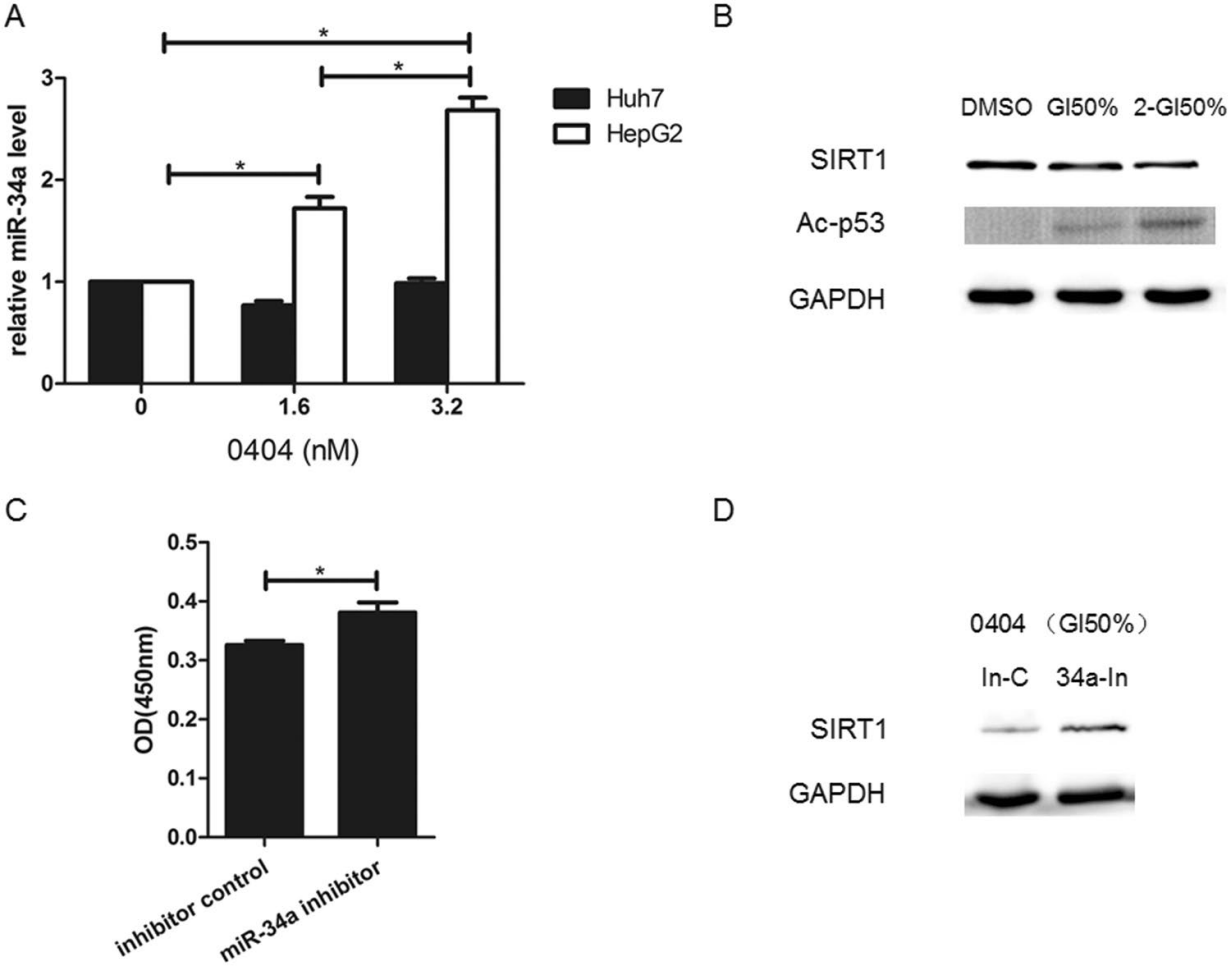
The anti-cancer effects of 0404 was closely related to miR-34a. (**A**) 0404 increased miR-34a level in a concentration-dependent manner in HepG2 cells (p53 WT). (**B**) Expression of SIRT1 protein and acylation of p53 after 0404 treatment. (**C**) MiR-34a inhibitor attenuated the inhibitory effects of 0404 in HepG2 growth when compared with the NC inhibitor. (**D**) SIRT1 expression significantly elevated in 0404 + miR-34a inhibitor group.

We further explored whether miR-34a was involved in p53-mediated anti-cancer effect of 0404. As shown in Fig. 6A, 0404 was able to increase the miR-34a expression in a concentration-dependent manner in HepG2 cells (p53 WT). Figure 6B showed that SIRT1 protein expression was down-regulated with the increase of 0404 concentration, while the level of acylation p53 was up-regulated with the increase of 0404 concentration. However, in Huh7 cells (p53 Mut), 0404 induced no down-regulation of SIRT1 and up-regulation of miR-34a (data not shown).

After administration of miR-34a inhibitor, the inhibitory effects of 0404 in HepG2 growth were attenuated compared with the negative control (NC) inhibitor (Fig. 6C). Moreover, SIRT1 expression was significantly elevated in 0404 + miR-34a inhibitor group, as compared with 0404 + NC inhibitor group (Fig. 6D).

Taken together, our results suggested that 0404 contributed to the expression of miR-34a in hepatoma cells, and such process was largely depending on p53. Besides, 0404 may inhibit HCC growth by forming a p53/miR-34a/SIRT1/Ac-p53 positive feedback loop.

## Discussion

As is known to all, the impedance-based time-dependent cell response profiles may be similar in compounds with similar activity. The compounds were classified on a basis of similar TCRP. Up to now, many distinct profiles such as antimitotic, cytostatic, and glucocoticoids have been discovered^8^. It’s worth noting that we also characterized a new DNA damaging compound 0404 using TCRP technique.

In this study, the anti-cancer effects of 0404 were investigated including *in vitro* and *in vivo*, together with the potential molecular mechanisms. *In vitro* study showed 0404 induced reduction of HCC cell viability in a concentration-dependent manner. Meanwhile, HepG2 cells with wild-type p53 were more sensitive to 0404 than Huh7 cells with mutant p53. There was little growth inhibition on Chang liver cell line after 0404 treatment, implying 0404 caused no cytotoxicity on the nontumorigenic human hepatocytes (data not shown). Besides, 0404 caused apoptosis of HepG2 cells in a concentration-dependent manner rather than in Huh7 cell, which further clarified that 0404 was more effective in treating wild-type p53 hepatic carcinoma. *In vivo* study revealed that 0404 could block the tumor progression in HepG2 bearing nude mice. Molecularly, 0404 up-regulated p53 and Ac-p53 expressions and down-regulated SIRT1 level, and the expression of TRAF2, which was known to regulate p53 expression by ubiquitin modifications^10^, was also decreased by 0404-treatment. Additionally, our findings suggest that these effects may be related to the up-regulation of miR-34a. Thus, 0404 may serve as a novel strategy for the treatment of HCC.

This discovery of 0404 as the agent against HCC may contribute to the improvement of the current therapeutic strategies. Nowadays, the majority of patients show poor response to HCC after administration of available chemotherapeutic drugs^14^. For instance, sorafenib is considered as a forefront agent for treating HCC^15^. Unfortunately, mild overall survival was reported, and many patients showed progressions of the disease^16^. Other agents such as sunitinib have been used in the treatment of HCC in many studies. Nevertheless, these drugs failed to display obvious superiority to sorafenib^17^. Thus, it is necessitous to identify effective treatment options with less side effects. The results in our study indicated that 0404 induced apoptosis effectively in HCC cells at a nM concentration, while, it caused no toxicity to nontumor hepatocytes Chang cells at that concentration. However, the comparison of anti-cancer effects and side effects between sorafenib and 0404 in *in vivo* yet to be verified.

The expression of p53 protein is activated in DNA-damaging process^18^. Ser15 phosphorylation of p53 mediated by ATM or ATR^19^ impairs the binding of mouse double minute 2 homolog (MDM2) to p53, which promotes accumulation and activation of p53 in response to DNA damage^20^. Up to now, many studies reveal p53 is closely related to the biological roles of miRNAs^21^, a class of single-stranded noncoding RNAs^22^ acting as tumor suppressors or oncogenes^23, 24^. In the p53 network, miRNAs are reported to regulate the p53 activity, and in turn p53 also participates in the regulation of miRNAs. To be exact, miRNAs involved in the p53 activity through targeting the 3′UTR of its mRNA directly or by suppressing the corresponding regulators. Alternatively, p53 could regulate the transcription and maturation of a cluster of miRNAs directly or indirectly, which consequently triggers many cellular responses. On this basis, it assumed that a feedback loop may form between p53 and miRNAs in the p53 network^21^. Among the miRNAs, miR-34 family, usually silenced in many tumors, has been identified as the most common p53-induced miRNAs^25, 26^. Our results verified the presence of p53/miR-34a/SIRT1/Ac-p53 positive feedback loop and approved such loop was closely related to the anti-cancer effects of 0404.

In conclusion, 0404 could inhibit the growth of liver cancer cells largely through modulating p53 associated pathway. Within the pathway, miR-34a is a crucial component of a feedback loop, which not only promotes the p53-related apoptosis signal but also contributes to the susceptibility of cancer cells to 0404. What’s more, we speculate that the mechanism of 0404 may be more various contrasted to other chemotherapeutic drugs. In future, further studies are necessary for investigating more anti-cancer mechanisms of 0404.

## Materials and Methods

### Clinical tissue specimens

Five pairs of matched HCC specimens and adjacent non-cancerous liver samples were got from HCC patients at the First Affiliated Hospital of School of Medicine, Zhejiang University. All patients signed the informed consent. The study protocols were approved by the Ethical Committee of the First Affiliated Hospital of Zhejiang University. All methods involving human tissue samples were performed in accordance with the relevant guidelines and regulations.

### Cell lines and drugs

The American Type Culture Collection offered the cell lines HepG2, Huh7, Chang, H292, A549 cells in the study. 0404 was synthesized in ACEA Biosciences. 5-Fluorouracil (5-FU) and Nutlin-3 were acquired from Sigma.

### Real Time Cellular Analysis

The xCELLigence DP device (Roche Diagnostics, Germany) was used to monitor cell proliferation in real-time. The 96 well electronic microtiter plates (Roche Diagnostics, Germany) were used to culture H292 cells (5 × 10^5^), and the cells were treated with various concentrations of 0404 or Nutlin or 5-FU, respectively. The xCELLigence system was used to measure all cells for 96 h according to the instructions. A programmed signal detection was used to measure the cell density in quadruplicate every 30 min. The RTCA software (version 1.2) from Roche Diagnostics was used for collecting and analyzing data.

### Cell viability assay

The cell counting kit-8 (Dojindo Laboratories, Japan) was used for measuring cell viability. Briefly, 96-well plates were used for culturing cells (5 × 10^3^ per well) in triplicate and cells were cultured with DMEM contained 0nM, 0.076 nM, 0.23 nM, 0.69 nM, 2.06 nM, 6.17 nM, 18.5 nM, 55.5 nM, 166.5 nM, 500 nM of 0404 for 72 h. At the time point, the cultures were added with the cell counting kit-8 reagent according to the instructions. After 1 to 4 hours of incubation, microtiterplate reader (BioRad, USA) was used to test absorbance at 450 nm.

### Cell cycle analysis

Six-well plates were used to culture a cell suspension corresponding to 5 × 10^5^ per well. After incubated at 37 °C for 24 h, HepG2 or Huh7 cells were treated with 0404 of 0 nM, 1.6 nM, 3.2 nM for another 24 h. A cell-cycle staining kit (MultiSciences, China) was used to stain cells in accordance with the manufacturer’s instructions. BD Accuri^®^ C6 flow cytometer (USA) was used to detect the distribution of cell-cycle phases.

### Cell apoptosis analysis

6 well plates were used to culture HepG2 or Huh7 cells (5 × 10^5^ per well), and cells were treated with 0404 for 24 h. C6 Accuri^®^ flow cytometer (BD, USA) was used for measuring the number of apoptotic cells using Annexin V assay kit (BD, USA). The CFlow software (version 1.0.264.25, Accuri cytometers) was used to analyze the data.

### Transfection

MiRNA-34a inhibitor and control scrambled siRNA (Life Technologies, USA) were purchased from Shanghai Invitrogen, and then were transfected into HCC cells by Lipofectamine RNAiMAX (Invitrogen, USA) in accordance with the manufacturer’s directions. 24 h later, 0404 was added and the cells were incubated for another 24 h. Afterwards, cells were harvested for further analysis.

### Western blotting

RIPA lysis buffer (Beyotime Biotechnology, China) containing 1% Halt™ Protease and Phosphatase Inhibitor Cocktail (100×) (Thermo Fisher Scientific) was used to lyse cells. Pierce BCA Protein assay kit (Thermo Fisher Scientific, USA) was used to measure the protein concentration. The protein (40 μg) was separated by 10% SDS-PAGE and then transferred to PVDF membranes (Millipore, USA). The membrane was blocked in TBST containing 3% BSA and primary antibodies were incubated overnight at 4 °C. The primary antibodies were used as follows: anti-SIRT1, anti-p53, anti-Ac-p53, anti-p-p53, anti-p-Chk2, anti-p21, anti-TRAF2, anti-p-ATM, anti-Cyclin B1 (1:1000, Abcam) and anti-GAPDH (1:5000, Huabio). Subsequently, TBST was used to wash the membranes three times and the membranes were incubated at room temperature for 1 h with 1:3000 horseradish peroxidase conjugated anti-rabbit or mouse immunoglobulin G (Southern Biotechnology Associates, USA), followed by three washings in TBST. Amersham ECL Plus Western Blotting Detection Reagents (GE Healthcare) were used to visualize the specific bands with a ChemiScope 3300 Mini equipment (CLINX, China). The same membrane loaded with GAPDH served as the control.

### Quantitative real-time PCR

Trizol (Invitrogen, USA) was used to isolate RNA. PrimeScript^®^ RT reagent Kit (Takara, Japan) was used to transcribe total RNAs into cDNAs. ABI Prism 7900 (Applied Bio systems, USA) was used to perform relative quantitative real-time PCR using the SYBR Premix ExTaq kit (TAKARA, Japan). In order to quantify the miRNA expression levels, comparative cycle threshold (Ct) method was used. The internal control was U6 small nuclear RNA. Relative quantification (2^−ΔΔCt^) was used to calculate the relative expression levels. The primers used in the study were designed based on published miRNA sequences of miRNA-34a, U6 (Life Technologies, USA).

### Transcriptome and MiRNA Microarray Profiling

HepG2 cells were treated with GI50% concentration of 0404 for 12 h. MirVana™ miRNA Isolation Kit (Invitrogen, USA) was used to extract total miRNA and Trizol (Invitrogen, USA) was used to isolate RNA. The miRNAs’ and RNAs expression profiling were performed by Affymetrix microRNA 3.0 array (Affymetrix, USA) and GeneChip^®^ PrimeView™ Human Gene Expression Array (Affymetrix, USA) in accordance with the manufacturer’s instructions, respectively.

### *In vivo* HCC xenograft mouse model

6 weeks old male Balb/c nude mice, which were purchased from Zhejiang Academy of Medical Science, were used for animal experiments. All methods involving mice were carried out in accordance with relevant guidelines and regulations. All experimental protocols involving mice were approved by the laboratory animal center of the First Affiliated Hospital of Zhejiang University. HepG2-luci cells (5 × 10^6^) were inoculated into mice subcutaneously. 7 days later, these mice were randomized into two groups before 0404 treatment. 4 μmol/kg 0404 was injected intraperitoneally once a day for 25 days. Visualization of luciferase activity was detected bioweekly using the Lmazuone FM1024 equipment (Nippon Roper, Japan). After mice were anesthetized with isoflurane, D-luciferin potassium salt (122799, Perkin Elmer, EU) at 150 mg/kg was then intraperitoneally injected. The SlideBook™ 4.0 software (Intelligent Imaging Innovations, USA) was used for data analysis including quantification. At last, we sacrificed the mice and excised the tumors and the tumor weight and volume were recorded accordingly.

### Immunofluorescence and confocal analysis

A549 cells grown on glass coverslips were treated with 0404 for 3 h, 8 h and DMSO alone was used as control. Next, 4% paraformaldehyde was used to fix the cells for 30 minutes. Then 0.5% Triton X-100 was used to permeabilize cells (20 min) and 1% bovine serum albumin in PBS was used to block them for 1 hour. Cells were incubated overnight at 4 °C with a rabbit polyclonal antibodies against γH2A.X (Abcam). Then an Alexa Fluor 488-labeled secondary antibody (Invitrogen) was used to incubate with the cells at room temperature for 1 hour. Actin filaments were stained using Alexa Fluor^®^ 555-labeled phalloidin. The slides were counterstained with DAPI (Sigma) and mounted using 2% propyl gallate. A confocal microscope of Olympus and an Image-Pro Plus 5.0 software of Media Cybernetics were used to view and acquire images.

### Immunohistochemical staining

Xenografted tumor tissues were immunohistochemically stained with p53 (1:500 dilution; Proteintech). The tissue sections were incubated with the antibody at 4 °C overnight.

### TUNEL assay

TUNEL staining was used to analyze the apoptotic cells in xenografted tumor tissues and we used the *in situ* cell death detection kit (Roche Diagnostics, Germany) on the basis of the protocol.

### Statistical analysis

SPSS version 19.0 was used for the data analysis. The differences between groups were evaluated through conventional Student’s t-test. The data were presented as mean ± SD and we repeated our *in vitro* experiment at least three times. P < 0.05 was considered to be statistically significant.

## Electronic supplementary material

Supplementary Information
