# Supplementary material for: 0404 inhibits hepatocellular carcinoma through a p53/miR-34a/SIRT1 positive feedback loop
Source: Sci Rep. 2017 Jun 30;7:4396. doi: 10.1038/s41598-017-04487-x (PMC5493669; doi:10.1038/s41598-017-04487-x)

0404 inhibits hepatocellular carcinoma through a p53/miR-34a/SIRT1 positive feedback loop

Caixia Xia[1, 2, a], Liyan Shui[1, a], Guohua Lou[1], Bingjue Ye[1], Wei Zhu[1], Jing Wang[1], Shanshan Wu[1], Xiao Xu[3], Long Mao[3], Wanhong Xu[3], Zhi Chen[1], Yanning Liu [1, *], Min Zheng [1, *]

[1] The State Key Laboratory for Diagnosis and Treatment of Infectious Diseases, The First Affiliated Hospital of School of Medicine, Zhejiang University. Collaborative Innovation Center for Diagnosis and Treatment of Infectious Diseases, Hangzhou, China.

[2] Department of Infectious Diseases, Hangzhou First People's Hospital, Nanjing Medical University, No. 261 Huansha Road, Hangzhou, China.

[3] Hangzhou ACEA Pharmaceutical Research Co., Ltd., Hangzhou, China

[a] These authors contributed equally to this work

* Corresponding author

Address to corresponding author: The State Key Laboratory of Infectious Disease Diagnosis and Treatment, The First Affiliated Hospital of School of Medicine, Zhejiang University, Hangzhou 310003, China

Tel: 86-571-87236579

Fax: 86-571-87068731

E-mail: minzheng@zju.edu.cn, rainyliu2002@163.com

Short title: 0404 inhibits hepatocellular carcinoma

Supplementary Figure 1.

Different 0404 sensitivities displayed by 6 different cancer cell lines. 0404 was more effective in p53 wide-type cell lines than in p53 mutant cell lines.

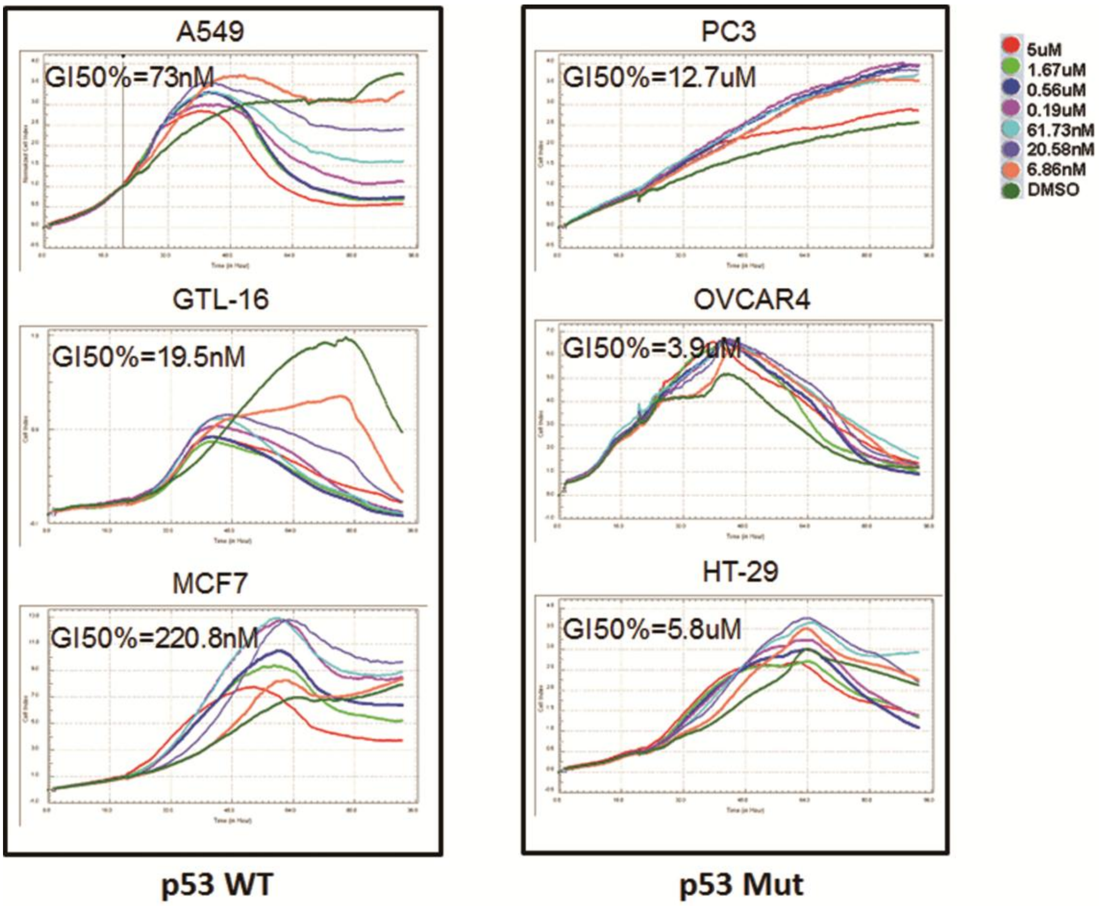

Supplementary Figure 2.

The full-length blots of Figure 2. (a) The full-length blots of Figure 2b. (b) The full-length blots of Figure 2c.

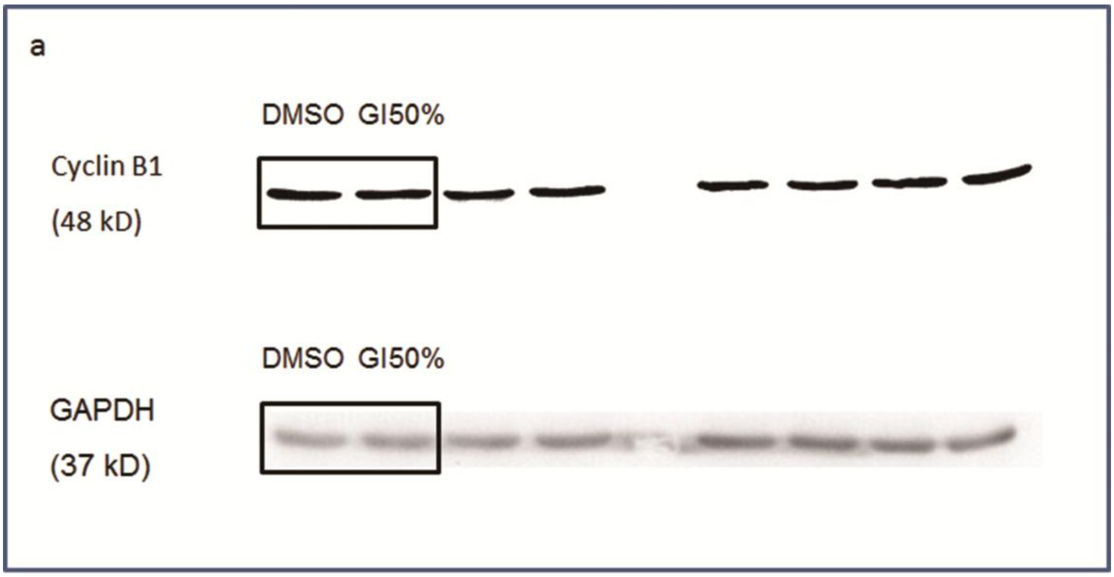

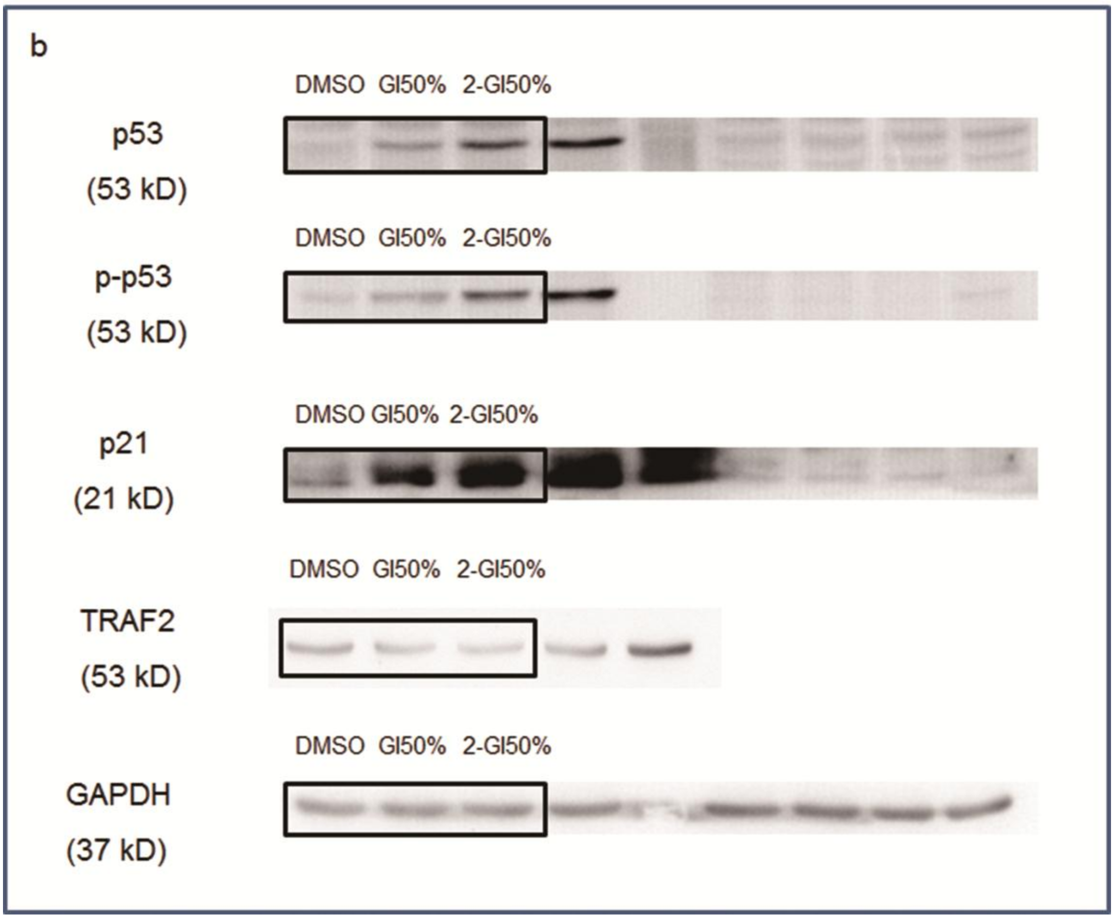

Supplementary Figure 3.
The full-length blots of Figure 5. (a) The full-length blots of Figure 5c.

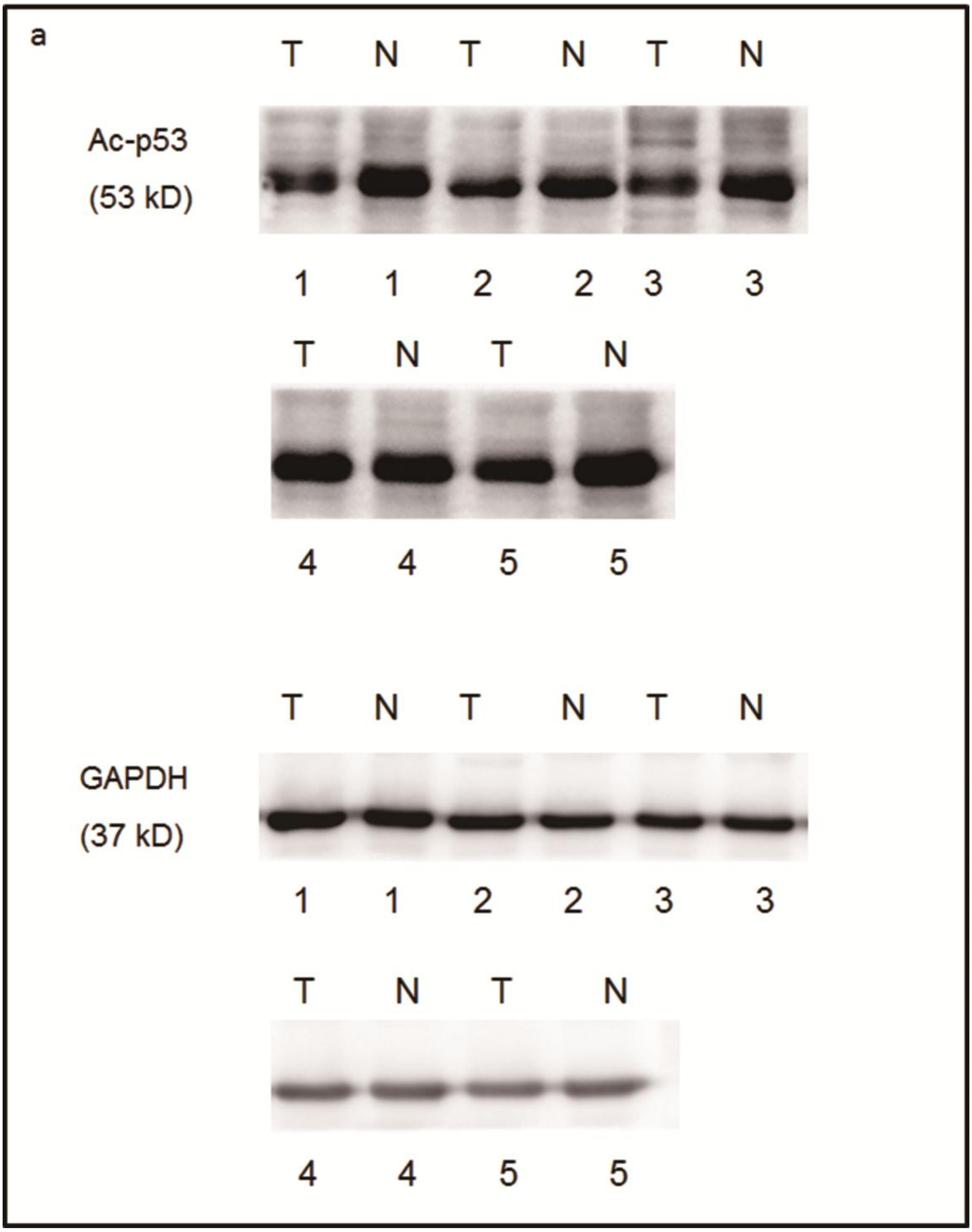

Supplementary Figure 4.

The full-length blots of Figure 6. (a) The full-length blots of Figure 6b. (b) The full-length blots of Figure 6d.

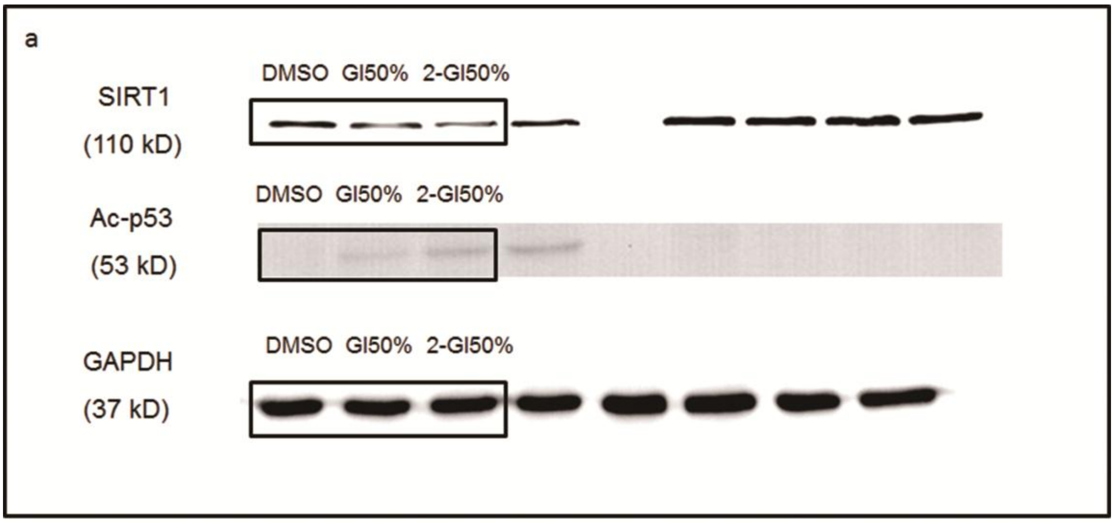

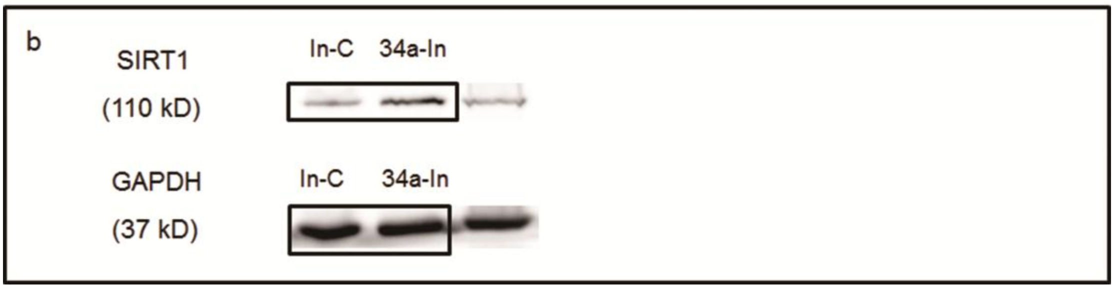

Supplement: Supplementary file 1 — Supplementary Information [file 41598_2017_4487_MOESM1_ESM.pdf]
